# Enhancing dosimetric practices through knowledge-based predictive models: a case study on VMAT prostate irradiation

**DOI:** 10.3389/fonc.2024.1320002

**Published:** 2024-01-17

**Authors:** Ahmed Hadj Henni, Ilias Arhoun, Amine Boussetta, Walid Daou, Alexandre Marque

**Affiliations:** ^1^ Radiation Oncology Department, Centre Frederic Joliot, Rouen, France; ^2^ Mohammed VI Polytechnic University, Ben Guerir, Morocco; ^3^ Oncology Department, Clinique Saint Hilaire, Rouen, France

**Keywords:** VMAT, prostate cancer, predictive models, geometric variables, treatment planning, knowledge-based planning

## Abstract

**Introduction:**

Acquisition of dosimetric knowledge by radiation therapy planners is a protracted and complex process. This study delves into the impact of empirical predictive models based on the knowledge-based planning (KBP) methodology, aimed at detecting suboptimal results and homogenizing and improving existing practices for prostate cancer. Moreover, the dosimetric effect of implementing these models into routine clinical practice was also assessed.

**Materials and methods:**

Based on the KBP method, we analyzed 25 prostate treatment plans performed using VMAT by expert operators, aiming to correlate dose indicators with patient geometry. The $D_{avg}^{Cav}\left(Gy\right)$ , $V_{45Gy}^{Cav}\left(cc\right),$ and $V_{15Gy}^{Cav}\left(cc\right)$ of the peritoneal cavity and the $V_{60Gy}\left(\%\right)$ and $V_{70Gy}\left(\%\right)$ of the rectum and bladder were linked to geometric characteristics such as the distance from the planning target volume (PTV) to the organs at risk (OAR), the volume of the OAR, or the overlap between the PTV and the OAR. In the second phase, the KBP was used in routine clinical practice in a prospective cohort of 25 patients and compared with the 41 patient plans calculated before implementing the tool.

**Results:**

Using linear regression, we identified strong geometric predictive factors for the peritoneal cavity, rectum, and bladder ( $R^{2}\;$ > 0.8), with an average prescribed dose of 97.8%, covering 95% of the target volume. The use of the model led to a significant dose reduction (Δ) for all evaluated OARs. This trend was most notable for $\text{Δ}V_{15Gy}^{Cav}=-171.5\text{ cc }\left(\text{p}=0.003\right)$ . Significant reductions were also obtained in average doses to the rectum and bladder, $\text{Δ}D_{avg}^{Rect}=\;-2.3\;Gy\;\left(\text{p}=0.040\right),$ and $\text{Δ}D_{avg}^{Vess}=\;-3.3\;Gy\;\left(\text{p}=0.039\right)$ respectively. Based on this model, we reduced the number of plans with OAR constraints above the clinical recommendations from 19% to 8%.

**Conclusions:**

The KBP methodology established a robust and personalized predictive model for dose estimation to organs at risk in prostate cancer. Implementing the model resulted in improved sparing of these organs. Notably, it yields a solid foundation for harmonizing dosimetric practices, alerting us to suboptimal results, and improving our knowledge.

## 1 Introduction

Intensity-modulated radiation therapy (IMRT) and volumetric-modulated arc therapy (VMAT) are standard techniques for treating prostate cancer, allowing for dose escalation to the planning target volume (PTV) with improved organ-at-risk (OAR) sparing of organs at risk (1, 2).

However, the quality of VMAT plans remains largely operator-dependent within most treatment planning systems (TPS). Variables such as the beam setup, creation of optimization volumes, desired dose objectives, optimization constraints, and balance-weighted priorities between the PTV and various OAR are critical factors affecting the final result.

This inverse planning approach usually requires considerable manual effort and skill to generate high-quality treatment plans. In particular, this process often takes several days between patient simulation and treatment initiation.

Dose constraints used in clinical centers are generally based on recommendations from expert organizations such as the Radiation Therapy Oncology Group (RTOG). These objectives have been determined based on studies including a large demographic database. While these protocols are often appropriate, for certain patients and geometric arrangements of the target volume and the OARs, they may sometimes be too broad or unattainable. Moreover, the training process for novices is protracted and complex, and their skills directly affects the quality of the proposed plan (3–5). Consequently, the lack of quantitative metrics available to physicians and physicists to assess whether an optimal dose distribution has been achieved may result in new calculation iterations or validation of suboptimal plans. Furthermore, this difference in the quality of treatment plans between planners or more widely between centers can have a significant therapeutic impact (6, 7).

The knowledge based planning (KBP) method represents a concept that guides planners by indicating achievable doses at the OAR while preserving the PTV coverage based on the patient’s anatomical parameters.

Related studies since 2008 (8) have demonstrated the ability of KBP methods to homogenize and improve practices, yield quality assurance for plans submitted for clinical validation, and serve as a training tool for novices. These improvements were based on the center’s experience in associating doses and predictive geometric variables.

Prostate cancer, a frequently treated tumor site, serves as an ideal context for implementing quality assurance and practice improvement methodologies (8). Most teams use the KBP method to predict a single dosimetric quantity to the rectum or bladder, often the mean dose. This study aimed to create and implement routine clinical empirical mathematical models predictive of particular doses to detect suboptimal plans and to identify the best practices for patients irradiated for prostate cancer. In this study, several indicators are proposed to assist with dosimetric planning. To the best of our knowledge, the peritoneal cavity has never been considered a reference structure for optimal dosimetry. In addition to rectal and bladder OAR, the peritoneal cavity was studied. In particular, V15 Gy and V45 Gy were found in prior work (9, 10) to be associated with grade 3 and acute toxicities in the peritoneal cavity and small intestine, respectively.

## 2 Materials and methods

First, the creation of mathematical models for the three important OAR in this location has been described: the peritoneal cavity, rectum, and bladder. The obtained models were directly integrated into the clinical routine using a script that calculated the expected doses based on the collected geometric variables. Second, the potential clinical impact of these models was assessed by comparing the overall dosimetric results before and after using this tool.

### 2.1 Patient selection, imaging, and treatment plans

We studied data from 91 patients treated with intensity modulation for prostate cancer at our center. The period covered was from April 2020 to January 2023.

The patients provided informed consent in accordance with the National Recommendations of the MR004 Regulation (CNIL). All the patients assumed a supine position, with standard equipment immobilization. CT (computed tomography) images (Somatom Definition AS20 RT, Siemens©) were acquired using a standardized protocol for the pelvis, empty rectum, and full bladder with a slice thickness of 1.5 mm. The upper anatomical limit of acquisition was the diaphragm, including the kidneys, and the lower limit extended to the mid-femurs.

An initial dose of 46 Gy in 23 fractions was delivered to PTV46, including the prostate, seminal vesicles, and lymph nodes. Subsequently, a dose of 30 Gy in 15 fractions was added to the PTV76, covering the prostate alone. The peritoneal cavity was delimited by the pelvic wall laterally, the posterior aspect of the abdominal wall anteriorly, the rectum and bladder below and the upper edge of the iliac bone above, excluding the CTVs, bladder, rectum and abdominal-pelvic muscles.

Treatment plans, based on two to three arcs of 6 MV photons at 600 MU/min, were calculated using the Eclipse TPS with the AAA.15.6 algorithm (Varian Medical Systems, Palo Alto, CA, USA) with Volumetric Modulated Arc Therapy.

The plan acceptance criteria involved coverage of 95% of the PTV by at least 95% of the prescribed dose, with a Dmax of< 107% (11). Dose constraints at the OAR were defined according to national and international recommendations (12). **Table 1** lists the dosimetric constraints to be achieved for each OAR for the sum of the two treatment plans.

**Table 1:** Template of OAR dose constraints used by dosimetrist for prostate VMAT planning (10).

OAR	Dose constraints
**Peritoneal Cavity**	Dmax< 54Gy
	V45Gy< 150cc
	V15Gy< 830cc
**Rectum**	V40Gy< 40%
	V60Gy< 50%
	V70Gy< 25%
	V75Gy< 5%
**Bladder**	V60Gy< 50%
	V70Gy< 25%
**Anal Canal**	V55Gy< 100%
**Right Femoral Heads**	V52Gy< 10%
**Left Femoral Heads**	V52Gy< 10%

In the context of our study, Paddick’s conformity index (13) and homogeneity index (11) were also calculated. The first index (PCI) describes the degree of conformity between the prescribed dose and target volume. The closer this parameter is to 1, the better the dose delivered to the target volumes. The homogeneity index of the dose distribution in the target volume is defined as HI = (D2% - D98%)/D50%, which should be as close as possible to 0.

The treatment plans were checked and validated by senior radiation oncologists and physicists. Prior to patient irradiation, usual quality controls were performed using an embedded portal imager to validate the treatment plan. Patients were then treated on a Truebeam MLC120 (Varian©) according to the center’s management protocol.

### 2.2 Parameters, models, and clinical implementation

#### 2.2.1 Predictive models

We created predictive models for the OAR dose based on a retrospective analysis of 25 patient plans calculated by expert operators. Volumetric information, i.e. the percentage overlap of the OAR with the target volume (V_OV(OAR/PTV)_/V_OAR_ (%)), and spatial information, i.e. the distance between the OAR and the PTV (Dist_OAR→PTV_ (cm)), were used to predict several dose indicators for the rectum, bladder, and peritoneal cavity using simple linear models.

For the rectum, $V_{60Gy}\left(\%\right)$ and $V_{70Gy}\left(\%\right)$ correlated with the ratio $V_{OV\left(Rect/PTV76\right)}/V_{Rect}\left(\%\right)$ . Similarly, bladder $V_{60Gy}\left(\%\right)$ and $V_{70Gy}\left(\%\right)$ correlated with $V_{OV\left(Blad/PTV76\right)}/V_{Blad}\left(\%\right)$ . The average dose of the peritoneal cavity, $D_{avg}^{Cav}\left(Gy\right)$ , was related to the distance between the center of mass of this structure and that of PTV46, $Dist_{Cav\to PTV46}(cm$ ). The V45Gy(cc) of this structure was related to the $V_{OV\left(Cav/PTV46\right)}/V_{Cav}$ . V15Gy (cc) was related to the product of $V_{OV\left(VCav/PTV46\right)}\times \;V_{Cav}$ .

#### 2.2.2 Comparison of treatment plans with and without the use of models

The developed models were used in routine clinical practice in a prospective cohort of 25 patients. The first step for the operator was to perform an initial optimization, considering the center’s dosimetric constraints for target volume coverage and OAR sparing. In the second step, various predictive volumes and distances were input into a script implemented by the physics team. Based on these geometric factors, the expected dose values for the three organs at risk, together with the associated 95% confidence intervals, were automatically calculated. By comparing the initial dosimetric results with the dose predictions for the rectum, bladder, and peritoneal cavity, the operator could reiterate the optimization as many times as necessary for the outcome to tend toward the predictions.

To evaluate the achieved dosimetric quality, these 25 new treatment plans were compared with 41 treatment plans calculated before the implementation of this tool.

#### 2.2.3 Statistical methods

The linear relationships between the geometric variables and dose indicators for the three OAR are presented graphically in the Results section, together with the associated coefficients of determination (R^2^). The significance of the comparison between the two plan samples, with and without the use of the model, was calculated using the nonparametric Mann–Whitney test. The results were associated with a p-value with a threshold value of 0.05, below which the difference was considered significant. The results are presented as the mean value (Mean) and associated standard deviation (SD). All statistical analyses were performed using the XLSTAT v2022 software.

## 3 Results

### 3.1 Results A: Predictive mathematical models for the rectum, bladder, and peritoneal cavity

**Figure 1:**
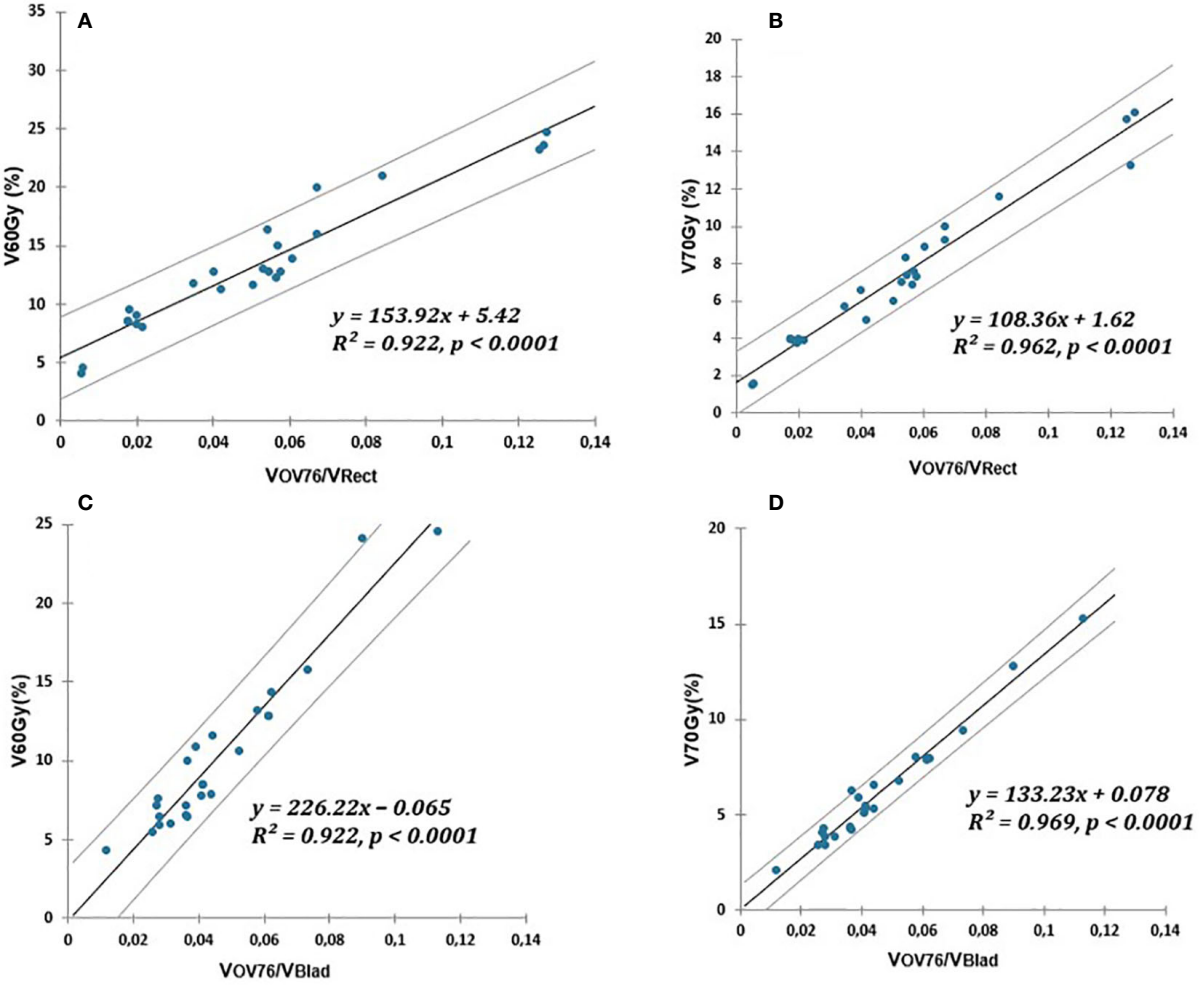
Linear relationship (solid lines) of V60Gy(%) and V70Gy(%) of rectum or bladder and PTV76 overlap normalized to OAR volume. **(A, B)** for the rectum. **(C, D)** for the bladder. The prediction uncertainties (95% confidence intervals) are represented by the dotted lines.

For the rectum and bladder, $V_{60Gy}\left(\%\right)$ and $V_{70Gy}\left(\%\right)$ correlated with the overlap between the OAR and PTV76 normalized to the OAR volume. All correlations were described by a linear regression of the type V_XGy_ (%) = $A*V_{OV\left(OAR/PTV76\right)}/V_{OAR}\left(\%\right)+B\left(\%\right)$ . All the results are summarized in **Figure 1**.

**Figure 2:**
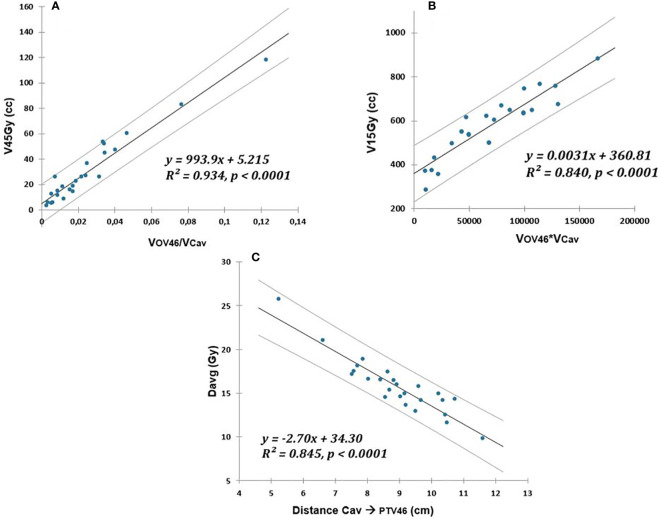
Linear relationship (solid lines) between V45Gy (cc), V15Gy (cc), and Davg (Gy) of peritoneal cavity and respectively: **(A)** overlap with PTV46 normalized to OAR volume, **(B)** overlap with PTV46 multiplied by OAR volume, **(C)** distance to PTV46. The prediction uncertainties (95% confidence intervals) are represented by the dotted lines.

The V45(Gy), V15(Gy), and $D_{avg}^{Cav}\left(Gy\right)$ of the peritoneal cavity were also correlated via a linear fit to the variables $\left(V_{OV\left(Cav/PTV46\right)}/V_{Cav}\right)$ , $\left(V_{OV\left(Cav/PTV46\right)}\times \;V_{Cav}\right)$ , and $(Dist_{Cav\to PTV46})(cm$ ), respectively. The peritoneal cavity models are illustrated in **Figure 2**.

### 3.2 Results B: Dosimetric evaluation of the model’s impact on clinical practice

After clinical implementation, 25 plans optimized using previously established models (WM) were compared with the 41 plans calculated prior to deployment (WOM).

#### 3.2.1 PTV46 coverage

**Table 2** details PTV46 coverage including prostate, seminal vesicles, and lymph nodes, with and without using the model. Recommended thresholds are also indicated. Target volume coverage for the second part of the irradiation (PTV76), consisting of a 30Gy boost in 15 fractions to the prostate alone, was not recorded in this study, as its simple shape did not present any particular difficulties in terms of dosimetric coverage.

**Table 2:** PTV46 coverage, prostate + seminal vesicles + lymph nodes, described by Paddick’s conformity index (PCI), homogeneity index (HI), dose received by 95% of the target volume (D95%), and maximum dose (Dmax) in the target volume.

Volume	Target Volume Predictors	Planning method		Thresholds
		Plans WM Mean(SD)	Plans WOM Mean(SD)	
**PTV76**	Volume (cm^3^)	131.9 (66.7)	141.4 (66.6)	
**PTV46**	Volume (cm^3^)	978.3 (39.2)	1002.4 (39.1)	
	PCI	0.813 (0.049)	0.847 (0.043)	> 0.7
	HI	0.074 (0.015)	0.074 (0.013)	Closest to 0
	D95%	97.83 (1.31)	97.77 (1.06)	> 95%
	Dmax (%)	107.40 (1.02)	107,13 (1.38)	< 107%

For each arc in the WM treatment plans, quality checks were performed by comparing the dose predicted by the portal dose image prediction (PDIP, Varian©) algorithm with the dose measured by the detector aS1200 EPID (Electronic Portal Imaging Device) on the accelerator. The average γ-index pass rate with threshold criteria of 2%/2 mm was 96.1% ± 4.3% using a dose threshold of 5%. All our plans (100%) met the validation criterion of a γ-index > 95% for thresholds of 3%/3 mm and a dose threshold of 5%.

Average target volumes, Mean(SD), PTV46 and PTV76 for the WM and WOM cohort were 978.3 cc (39.2 cc), 131.9 cc (66.7 cc), 1002.4 cc (39.1 cc), and 141.4 cc (66.6 cc), respectively (**Table 2**). The mean PCI for the two planning methods was above 0.7, which was defined as the lower acceptable limit. The mean(SD) PCI for the WM and WOM methods were not statiscally different and were 0.813(0.049) and 0.847(0.043) respectively. The mean homogeneity index was 0.074 in both cases. The doses received by 95% of the target volume were 97.83% of the prescribed dose using the model and 97.77% without the model. The Dmax in PTV46 was 107.40% and 107.13% for the WM and WOM treatment plans, respectively (**Table 2**).

#### 3.2.2 Evaluating doses to OAR

**Table 3** summarizes the dosimetric results for several OAR relevant to the validation of prostate cancer treatment plans, together with the dosimetric differences ${\text{Δ}}_{WM/WOM}$ (p-value) between the two methods. Significant improvements were observed for at least one dosimetric indicator in each of the three OAR modeled in this study, in favor of the WM method. This trend was the most notable for the peritoneal cavity on V15Gy (cc), with a decrease of 171.5cc (p = 0.003) and 12.2cc on V45Gy (cc) (p = 0.029). The mean doses to the rectum and bladder were reduced by 2.3 Gy (p = 0.040) and 3.3 Gy (p = 0.039), respectively. The comparison was also extended to OAR with no predictive pre-dosimetry dose indicators. Between the two methods, the final results either showed no significant differences (right and left femoral heads, sigmoid, and penile bulb) or were in favor of the use of the models in clinical practice (anal canal). A significant mean dose reduction of 3.6 Gy was observed in the anal canal when the WM method was used. For the penile bulb, the mean dose was 29.0 Gy when the operators used the predictive models versus 35.8 Gy without; however, this improvement was not statistically significant. Regardless of the method used (WM or WOM), there were no differences in the mean doses to the femoral head. For femoral heads, looser constraints were allowed during the optimization stage. This choice was made to limit anteroposterior irradiation of the rectum, bladder, and peritoneal cavity, given their more restrictive constraints compared to the femoral heads.

**Table 3:** Mean values and standard deviations, Mean(SD), for different dosimetric parameters of OAR for treatment plans with use of the model (WM) and without its use (WOM).

OARs	Dose Volume Predictors	Planning method		Difference
		Plans WM Mean(SD)	Plans WOM Mean(SD)	${\text{Δ}}_{WM-WOM}$ (p-value)
**Peritoneal Cavity**	Davg(Gy)	16.1 (6.0)	18.8 (6.3)	**- 2.6 (0.033)**
	V15Gy(cc)	497.7 (167.3)	669.2 (253.6)	**-171.5 (0.003)**
	V45Gy(cc)	33.2 (33.7)	45.4 (28.4)	**-12.2 (0.029)**
**Rectum**	Davg(Gy)	35.2 (4.8)	37.5 (4.0)	**-2.3 (0.040)**
	V50Gy(%)	19.9 (6.5)	23.4 (6.0)	**-3.5 (0.042)**
	V60Gy(%)	10.9 (4.9)	14.8 (4.1)	**-3.9 (0.001)**
	V70Gy(%)	5.4 (3.3)	7.7 (2.6)	**-2.4 (0.001)**
**Bladder**	Davg (Gy)	34.1 (7.2)	37.3 (4.4)	**-3.2 (0.039)**
	V60Gy(%)	10.4 (7.0)	11.1 (5.3)	-0.6 (0.253)
	V70Gy(%)	5.9 (3.8)	6.5 (3.3)	-0.6 (0.296)
**Anal Canal**	Davg(Gy)	14.6 (6.8)	18.2 (7.1)	**-3.6 (0.043)**
**Sigmoid**	Davg(Gy)	33.5 (6.9)	36.3 (5.2)	-2.8 (0.152)
**Right Femoral Heads**	Davg(Gy)	17.9 (4.0)	17.5 (3.6)	0.4 (0.723)
**Left Femoral Heads**	Davg(Gy)	17.1 (4.0)	17.1 (3.2)	0.0 (0.994)
**Penile Bulb**	Davg(Gy)	29.0 (22.6)	35.8 (22.4)	-6.8 (0.180)

Mean deviations ${\text{Δ}}_{\text{WM}-\text{WOM}}$ and p-values have been indicated in bold if statistically significant.

In terms of the overall quality of dosimetric plans, according to the calculations corresponding to the 41 patients performed using the WOM method, the planners were not able to meet the peritoneal constraint of 830 cm^3^ for 8 patients (19%). Among the 25 patients for whom the WM method was planned, two patients (8%) exceeded this limit.

## 4 Discussion

Over the past fifteen years (8), several studies have proposed dosimetric indicators to OAR based on KBP methodology.

For example, the work of Moore et al. (14), often cited in the literature, related the overlap between the PTV and OAR to the optimal mean dose received by the OAR in the case of prostate, head, and neck cancers by an exponential function. In addition, Powis et al. (15) confirmed these results in prostate cancer using the same methodology. Furthermore, Kang et al. (16) proposed simple linear regressions between the PTV and OAR overlaps in the heart and homolateral lung to predict the mean dose or different V_Gy_ (%) to the OAR in the case of left breast irradiation. For the same location, Tomatis et al. (17) used a more complex method of target volume expansion and overlapped it with OAR (18) to predict the mean dose to the heart. Recently, predictive models of dose distribution at different locations have been developed based on advanced deep learning strategies (19–21).

The aim of these pre-dosimetry predictions was to improve the quality of treatment plans and to accompany all planners, regardless of their level of expertise, in their optimization trials. Our objectives were identical.

Using simple linear models, we established a strong link between geometric predictors (**Figures 1**, **2**) and dosimetric indicators relevant to the rectum, bladder, and peritoneal cavity OAR. To the best of our knowledge, the multiplication of dosimetric indicators enabling effective quality control of the treatment plan has not yet been proposed. The contribution of this study, compared to previous work, was also to propose a more extensive list of geometric predictors. The models presented in this document can be easily adapted by other hospital settings, taking into account their own practices.

By comparing before and after the implementation of the KBP script, we could validate the positive impact of this methodology on our clinical practice. For the same level of target volume coverage (**Table 2**), OAR sparing was generally better. In the peritoneal cavity, V15Gy was reduced by more than 170cc on average.

Prior to the implementation of the WM model, it was difficult for operators (even experts) to predict the feasibility of the V15Gy constraint at the peritoneal cavity, given the low dose involved compared with the other constraints studied. This constraint was the most difficult to meet in many treatment plans. This constraint was the subject of extensive replanning before the model was implemented. Today, we have a predictive model on which we can base our discussions with physicians on feasibility and realization limits. This constraint was above the 830 cc threshold in 8% of the cases for the KBP method compared with 19% before implementation. Mean doses to the rectum and bladder were also significantly improved by 2.3 Gy and 3.2 Gy, respectively. When the comparison was extended to other OAR (**Table 3**), no deterioration was observed. In contrast, an improvement of 3.6 Gy was significant for the mean dose to the anal canal.

In 2020, Wall and Fontenot (22) also found an improvement in the mean dose for the rectum and bladder; however, they noticed that the KBP plans obtained were significantly more complex. According to this study, particular attention should be paid to the feasibility of these treatment plans for accelerators.

In our case, all pre-treatment patient quality controls performed using the portal dose image prediction (PDIP) met clinical validation thresholds (96.1% ± 4.3% with criteria set at 2%/2 mm). Notably, in the study by Wall and Fontenot (22), the average dose gain for the rectum and bladder was greater than 6 Gy, logically increasing the level of complexity of the calculated plans. Even though there was an increase in MU, the pre-treatment QA results were excellent and the increase in plan complexity of the plans were therefore deemed clinically acceptable.

However, these simple models require a substantial prior workload to determine the most relevant correlations between the predictive volumes and dosimetric indicators for each OAR. In addition, regular updates of these guide values are recommended (23) to improve the overall quality of treatment plans and adapt to possible changes in practice.

Our script has only been validated using our own data, similar to the majority of studies presented in the literature. This limits its direct use in other centers. A database based on the experiences of several teams can lead to more global predictive models. To achieve this objective, the AAPM (American Association of Physicists in Medicine) organized a challenge on this topic in 2020 (24).

Although planning systems (TPS) (25) can also offer automatic planning tools based on KBP, our methodology, which is a simple and cost-effective tool for clinical use, does not compete with commercial solutions. The strength of the methods presented in this manuscript are in their simplicity, as we create simple linear models based on geometric characteristics of the target, OARs and their relation to each other. Nevertheless, our main objective of improving our practices while preserving the expertise of our planners has been achieved. Subsequently, commercial solutions will be used by our teams to improve the overall planning time.

The next steps in our work will involve applying this planning strategy to other sites, particularly, breast cancer (16), given the large number of patients treated at our center. This tool may also be useful for treating head and neck cancers (14).

While our study successfully demonstrates the efficacy of knowledge-based predictive models in improving dosimetric practices for VMAT prostate irradiation, several intriguing questions and opportunities for future research emerge. Firstly, delving deeper into the impact of these models across diverse patient populations and treatment modalities could provide a more nuanced understanding of their generalizability. We propose a method that each center can implement with their own practices. It should be noted that the geometric indicators, distance between target volume and OARs or overlap between both, used in this work are intuitive and may be more generalizable by expanding our database or integrating those of other centers. The prior creation of a software interface to centralize this data and automate our methodology would facilitate this objective. This work, in collaboration with a school of computer science, is in progress.

Additionally, exploring the integration of advanced machine learning techniques or expanding the application of predictive models to other cancer sites, as briefly mentioned in the conclusion, holds promise for further enhancing treatment planning accuracy. Furthermore, longitudinal studies assessing the long-term clinical outcomes of patients treated with KBP-guided plans could provide valuable insights into the sustained benefits of this approach. By addressing these aspects, future research can continue to refine and expand the implementation of knowledge-based planning in radiation therapy, ultimately advancing the field.

## 5 Conclusions

In the context of prostate cancer treatment, this investigation has revealed strong linear correlations between geometric variables and dose indicators pertaining to the rectum, bladder, and peritoneal cavity. Our predictive models, based on clinical experience, have proven instrumental in guiding operators during treatment plan optimization. Their implementation in routine clinical practice has led to a significant reduction in doses to the rectum, bladder, and peritoneal cavity, without any deterioration in target volume coverage. Generally, these predictive models have strengthened the quality of our treatment plans, homogenized our practices, and offered valuable warning thresholds for potential suboptimal outcomes.

## Data availability statement

The original contributions presented in the study are included in the article/supplementary material. Further inquiries can be directed to the corresponding author.

## Author contributions

AH: Conceptualization, Formal analysis, Investigation, Methodology, Software, Supervision, Validation, Writing – original draft, Writing – review & editing. IA: Conceptualization, Formal analysis, Investigation, Methodology, Software, Validation, Writing – review & editing. AB: Investigation, Software, Validation, Writing – review & editing. WD: Investigation, Software, Validation, Writing – review & editing. AM: Conceptualization, Methodology, Validation, Writing – review & editing.
